# Genome Sequences of *Corynebacterium pseudotuberculosis* Strains 48252 (Human, Pneumonia), CS_10 (Lab Strain), Ft_2193/67 (Goat, Pus), and CCUG 27541

**DOI:** 10.1128/genomeA.00869-14

**Published:** 2014-09-04

**Authors:** Othilde Elise Håvelsrud, Henning Sørum, Peter Gaustad

**Affiliations:** a Department of Microbiology, Oslo University Hospital, Oslo, Norway; b Faculty of Veterinary Medicine and Biosciences, Norwegian University of Life Sciences, Oslo, Norway

## Abstract

Here we report the genome sequencess of four *Corynebacterium pseudotuberculosis* strains. These include a strain isolated from a patient with *C. pseudotuberculosis* pneumonia (48252), a strain isolated from pus in goat (Ft_2193/67), a laboratory strain originating from strain Ft_2193/67 (CS_10), and the draft genome of an equine reference strain, CCUG 27541.

## GENOME ANNOUNCEMENT

*Corynebacterium pseudotuberculosis* is an important animal pathogen, primarily in sheep and goat but also in other ungulates like horse and cow (1). Human infections are rare and usually in the form of granulomatous lymphadenitis (2, 3). One previous case of pulmonary infection has also been reported (4). Here we present the complete genomes of a goat strain (Ft_2193/67), a lab strain originating from strain Ft_2193/67 (CS_10), and a strain isolated from a patient with pulmonary infection (48252). We also present the draft genome of the equine reference strain CCUG 27541. To our knowledge, strain 48252 is the first whole-genome sequenced human *C. pseudotuberculosis* pneumonia strain. Together with the other sequenced genomes, the whole-genome-sequence of this strain presents a good opportunity for further investigation into *C. pseudotuberculosis* virulence.

DNA was extracted using the DNeasy blood and tissue kit (Qiagen), following the protocol for Gram-positive bacteria. The DNA was purified and concentrated using Amicon Ultra-0.5 mL 30-K centrifugal filters for DNA purification and concentration (Merck Millipore). The DNA quality was assessed by agarose gel electrophoresis combined with concentration and 260/280 measurements on a NanoDrop 2000 spectrophotometer (Thermo Scientific). The DNA was sequenced and assembled at the Norwegian Sequencing Centre (http://www.sequencing.uio.no/). The library was prepared according to the Pacific Biosciences 10-kb library preparation protocol. Size selection of the final library was conducted with BluePippin (Ampure Beads for strain CCUG 27541). Sequencing was done on a Pacific Biosciences RS II instrument using P4-C2 chemistry. Two silencing mediator of retinoic acid and thyroid hormone receptor (SMRT) cells were used for each strain. A total of 96,456 reads with an average read length of 4,677 bp were generated for strain 48252, 7,7683 reads of 4,664 bp on average for strain CS_10, 92,607 reads of 4,859 bp on average for strain Ft_2193/67, and 89,570 reads of 2,491 bp on average for strain CCUG 27541. The reads were assembled with HGAP v. 3 (Pacific Biosciences), resulting in one continuous contig for each of strains 48252, CS_10, and Ft_2193/67. The Minimus2 software (Amos package) was used to circularize the contigs from these strains. Assembly of strain CCUG 27541 resulted in five contigs. The two shortest contigs had considerably lower coverage (8 and 34) than the other three (99–120) and were excluded from further analyses. The three remaining contigs had a combined length of 2,379,417 bp and a GC content of 52.10%. The circularized contigs of strains 48252, CS_10, and Ft_2193/67 consist of 2,338,139 bp (average coverage 150), 2,338,144 bp (average coverage 130), and 2,338,300 bp (average coverage 151), respectively. All three genomes have a GC content of 52.19%.

The genomes were automatically annotated at the RAST server (v. 4.0) (http://rast.nmpdr.org/ [5]) using default options. The genomes contained 2,227, 2,232, 2,242, and 2,240 potentially protein-encoding genes for strains 48252, CS_10, Ft_2193/67, and CCUG 27541, respectively. Twelve rRNAs and 49 tRNAs were detected in each of strains 48252, CS_10, and Ft_2193/67. In the draft genome of strain CCUG 27541, 18 rRNAs and 51 tRNAs were detected.

### Nucleotide sequence accession numbers.

The genomes have been deposited in DDBJ/EMBL/GenBank under the accession numbers CP008922, CP008923, CP008924, and JPJB00000000. The versions described in this paper are the first versions, CP008922.1, CP008923.1, CP008924.1, and JPJB01000000.
